# Sepia, a taxonomy oriented read classifier in Rust

**DOI:** 10.21105/joss.03839

**Published:** 2021

**Authors:** Henk C. den Bakker, Lee S. Katz

**Affiliations:** 1 Center for Food Safety, University of Georgia, Griffin, GA, USA; 2 Enteric Diseases Laboratory Branch (EDLB), Centers for Disease Control and Prevention, Atlanta, GA, USA

## Abstract

Here we present Sepia, a fast and accurate read classifier. It is implemented in Rust, has the ability to switch between different taxonomies, can detect inconsistencies in taxonomies, and can estimate similarities between organisms in a query dataset and reference sequences in the index.

## Statement of need

Bioinformatics tools to infer the taxonomic composition of sets of biological sequences are quintessential for taxonomic profiling and contamination checking. A variety of tools have been developed to accomplish taxonomic profiling and these tools can be roughly classified into two categories: (i) read alignment based tools such as MIDAS (Nayfach et al., 2016) and MetaPhlan (Segata et al., 2012), which map reads against a database of reference sequences or taxon specific marker sequences, and (ii) read classification based tools, such as Kraken (Wood & Salzberg, 2014), Kraken2 (Wood et al., 2019) and Centrifuge (Kim et al., 2016). Many read classifiers use a least common ancestor (LCA) approach where reads in common among sibling taxa get assigned to the rank and taxonomy in common with those taxa; e.g., a kmer in common with both Escherichia and Salmonella, both members of the family Enterobacteriaceae, would be elevated to be associated in a database with the higher order taxon Enterobacteriaceae instead. While all these tools heavily rely on taxonomies, changing the taxonomies (e.g., correcting wrong placements of accessions or adapting a novel taxonomy) is not easily done. In response, we wrote Sepia, a taxonomic read classifier to address rapidly changing developments in taxonomy (e.g., the genome-based GTDB taxonomy (Parks et al., 2018)) and algorithm development. There are three areas where Sepia directly addresses issues needing improvement: (i) taxonomy, (ii) classification accuracy, and (iii) the ability to perform fast batch classification for multiple datasets.

### Taxonomy

Taxonomic read classifiers that use an LCA approach work on the assumption that biological taxonomies reflect evolutionary relationships, thus making it possible to use taxonomies as a predictive framework in read classification. Inclusion of artificial taxa that are not supported by a genome-based phylogeny (e.g., garbage bin taxa or taxa with an unknown placement ‘incertae sedis’) or artifactual errors (e.g., a genus placed erroneously in the wrong family in some of but not all of the accessions in the taxonomy) have a deleterious effect on the accuracy LCA based classification algorithms, potentially leading to some taxa not being classified at all or consistently classified as the wrong taxon. To address these issues Sepia uses human-readable taxonomy strings as input. While building the index putative ambiguities or inconsistencies in the taxonomy are flagged and logged to a file for the user to address.

### Sequence similarity and classification accuracy

**Figure 1:**
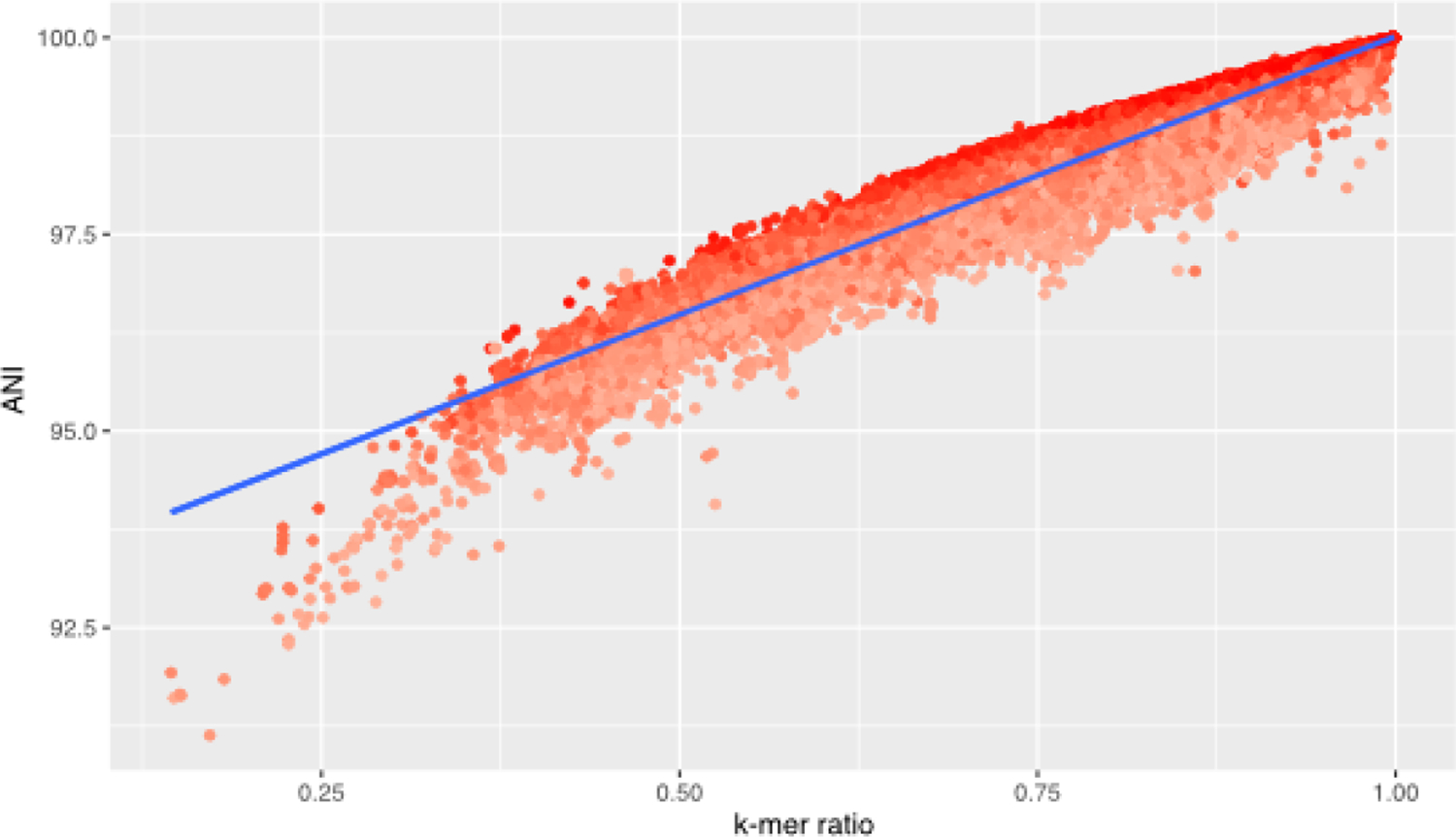
Correlation of Average Nucleotide Identity (ANI) as inferred by fastANI with minimizer-based estimation of k-mer similarity (p-value: « 0.001, Multiple R-squared: 0.96)

Read classifiers tend to miss- or overclassify reads, especially in situations where a lot of reads represent a taxon that is not present in the indexed reference dataset. To address this issue Sepia records the per read k-mer ratio, which is the ratio of k-mers supporting the proposed classification versus the total number of k-mers used for the classification. The average k-mer ratio highly correlates to the Average Nucleotide Identity (ANI) between reference strains in an index and strains in a query dataset (Figure 1), and low k-mer ratios can be used to remove over- or misclassified reads after classification.

### Batch classification

While the process of taxonomic read classification is usually fast, the time needed to load a large index into RAM (e.g., 47,894 accessions for reference sequences of GTDB rs202 requires a minimum of 98 Gb of RAM) can take longer than the actual classification process. When used repeatedly on a batch of sequence datasets, this can make the use of a read classifier time prohibitive. To overcome this time expensive hurdle, a batch classify function is included in Sepia; a user generated file containing sequence datasets for multiple samples is used as input, the index is read only once into RAM and subsequently the sequence data for the individual samples are classified.

## Implementation

Sepia is written in the Rust programming language. The k-mer or minimizer index is an implementation of the compact hash table described by (Wood et al., 2019). Briefly, this hash table consists of a fixed array of 32 bits hash cells to store key-value pairs with a generic hash function (i.e., murmurHash3 (Appleby, 2011) in Kraken 2 and Sepia) and a load factor of 70% for collision resolution, thus needing considerably more space than key-value pairs. Alternatively, the user can choose to use an experimental index with a perfect hash function (Limasset et al., 2017) as implemented in Rust (https://github.com/10XGenomics/rust-boomphf). A perfect hash function maps a set of actual key values to the table without any collisions, thereby potentially decreasing the space requirement of the hash table compared to the compact hash table of Kraken 2. Next, taxonomy information is encoded into unsigned 32 values such that higher order taxa always have a lower value than lower order taxa, allowing for rapid set operations to infer LCAs for a set k-mers or minimizers within a single read or read pair. There is no limit to the number of taxonomic levels in a taxonomy string. This allows for the user to combine different taxonomies for different taxonomic domains (e.g., NCBI viral taxonomy combined with GTDB taxonomy for Archaea and Bacteria). The unsigned 32 encoded taxonomy is compactly stored in a directed acyclic graph, the direction being from child to ancestral node. This allows for a rapid look-up of lineages and generation of sets for LCA inference. Upon completion Sepia produces two files; (i) a file with the per read or read pair classification, and (ii) a summary file, reporting the read count per taxon, the average k-mer ratio and the total length of all reads classified as a specific taxon. Using simulated and real datasets we found Sepia to be very similar in performance to Kraken 2; We found not significant differences in sensitivity and specificity of read classifications, however, we found Kraken 2 to be between 30% and 15% faster for Illumina short read datasets (HiSeq 125bp paired-end and MiSeq 300bp paired-end, respectively), however Sepia and Kraken 2 classified reads at a similar speed for an Oxford Nanopore dataset (NCBI SRA accession SRR15372305).
